# Laser SLAM Matching Localization Method for Subway Tunnel Point Clouds

**DOI:** 10.3390/s25123681

**Published:** 2025-06-12

**Authors:** Yi Zhang, Feiyang Dong, Qihao Sun, Weiwei Song

**Affiliations:** 1 School of Geodesy and Geomatics, Wuhan University, Wuhan 430072, China; yzhang@sgg.whu.edu.cn (Y.Z.); qihao_sun@huace.cn (Q.S.); 2 GNSS Research Center, Key Laboratory of Luojia of Hubei Province, Wuhan University, Wuhan 430072, China

**Keywords:** SLAM, subway tunnels, feature extraction, registration

## Abstract

When facing geometrically similar environments such as subway tunnels, Scan-Map registration is highly dependent on the correct initial value of the pose, otherwise mismatching is prone to occur, which limits the application of SLAM (Simultaneous Localization and Mapping) in tunnels. We propose a novel coarse-to-fine registration strategy that includes geometric feature extraction and a keyframe-based pose optimization model. The method involves initial feature point set acquisition through point distance calculations, followed by the extraction of line and plane features, and convex hull features based on the normal vector’s change rate. Coarse registration is achieved through rotation and translation using three types of feature sets, with the resulting pose serving as the initial value for fine registration via Point-Plane ICP. The algorithm’s accuracy and efficiency are validated using Innovusion lidar scans of a subway tunnel, achieving a single-frame point cloud registration accuracy of 3 cm within 0.7 s, significantly improving upon traditional registration algorithms. The study concludes that the proposed method effectively enhances SLAM’s applicability in challenging tunnel environments, ensuring high registration accuracy and efficiency.

## 1. Introduction

As one of the main modes of transportation for urban commuting, the subway relieves traffic pressure and reduces environmental pollution. The stability of its operation is very important. In subway tunnels, due to blocked communication and weak or missing GNSS (Global Navigation Satellite System) signals, when the subway is running, the traditional positioning method of GPS (Global Positioning System) combined with inertial measurement unit (IMU) will fail, and the safety of normal operation cannot be guaranteed. Other sensors are needed for auxiliary positioning. In recent years, with the development of the basic theory and application of SLAM technology, the environment-aware positioning function of SLAM has been gradually applied as a core technology to the auxiliary positioning modules of various vehicles and mobile robots. The LOAM (Zhang et al., 2014), is one of the most classic 3D SLAM systems [1]. The algorithm divides SLAM into two parts: front-end and back-end. The former achieves high-frequency but low-precision laser mileage. The latter completes the low-frequency but high-precision map creation work, and the two are combined into a high-precision and low-computational SLAM algorithm, which lays the foundation for 3D laser SLAM. In view of the excellent performance of LOAM, much follow-up work is carried out on its basis, focusing on solving some challenging problems in extreme or special scenarios. Based on LOAM, Shan et al. (2018) proposed the LeGo-LOAM, which increased the constraints of ground point information and improved the operation efficiency [2]. It added a loopback detection module in the back-end optimization, which further reduced the cumulative drift error. Since the advent of solid-state lidar, some scholars have also studied its characteristics. Fu Zhang et al. (2019) first proposed the LOAM-Livox algorithm based on Livox solid-state lidar [3]. The algorithm redefined the feature extraction method of point clouds and proposed a “bad point” screening assisted by spatial constraints combined with reflection intensity information in the fourth dimension. In addition to the strategy, the robustness of the system was enhanced, and the closed-loop detection module was also added in the back end to ensure the low drift and high precision of the entire system.

With the development of multi-sensor fusion, sensors such as IMU and visual cameras can complement lidar data to further improve the accuracy of laser SLAM. Based on LeGo-LOAM, Shan et al. (2020) proposed LIO-SAM [4], which tightly coupled lidar and IMU, using IMU data for correcting point cloud motion distortion. It had better robustness to fast rotating scenes. The FAST-LIO (Xu et al., 2021) tightly coupled the IMU data with the laser point cloud, and optimized the Kalman filter formula of the IEKF (Iterated Extended Kalman Filter), thereby speeding up the inversion operation and improving the overall operating efficiency [5]. On this basis, FAST-LIO2 (Xu et al., 2022) did not use line or surface features, but used the global point cloud for processing and maintained the map through an incremental KD tree (K-dimensional tree) structure [6]. In addition to an efficient nearest neighbors search, the new data structure also supported incremental map updating (i.e., point insertion, down-sampling, point deletion) and dynamic balancing with minimal computational cost. VLOAM (Zhang et al., 2015) established the VO (Visual Odometry) and the LO (Laser Odometry) at the same time [7]. VO operated at high frequency, obtaining the depth of part of the visual feature points through the laser, and the output poses were integrated to provide prior information for the estimation of transformation between frames, while LO performed low-frequency estimation of transformation between frames and frame-to-map pose estimation. Finally, it fused the pose which VO output as the final pose result, realizing the complementarity of camera and lidar data. VIL-SLAM (Shao et al., 2019) integrated three pieces of sensor information: vision, IMU, and laser. First, the initial value of the pose was estimated through the VIO (Visual-IMU Odometry) module, and then the estimation result was used to assist the laser scan matching [8] and the high-fidelity 3D point cloud was accumulated to form an accurate map. Finally, the hybrid ICP was used. The bag-of-words model performed closed-loop detection, which improved the robustness of the system. On the basis of LeGo-LOAM, Shan et al. (2021) proposed LVI-SAM [9], which also fused three types of data, and divided the system into VINS (Qin, 2018) and LINS (Qin, 2020) [10,11]. The two subsystems were tightly coupled, and the VINS was initialized with the estimation of the LINS and used lidar points to provide depth to visual features to improve accuracy. At the same time, the LINS used the estimation of VINS as the initial value to match adjacent frames. LVI-SAM can still work when one of the subsystems fails, which greatly improves the robustness of the system in scenes lacking textures or features.

Most of the above methods follow the line-surface feature matching method for motion estimation. Some scholars try to use other point cloud registration methods, such as the following: Moosmann (2011) used the classic ICP (Iterative Closest Point) in the SLAM system [12]. Koide (2019) used the surface element (surfel) model to construct the point cloud map [13]. And the NDT (Normal Distribution Transformation) was used to realize the registration of points to the map. Behley (2018) proposed a Surfel-based SLAM method, which used surfel-ICP to register points in the system [14]. Droeschel (2017) proposed a multi-resolution raster map representation method and used a probabilistic point set registration algorithm to match point clouds [15]. The intensity of the point cloud can reflect the surface material of the target, which can help the system to perceive the environment more comprehensively.

However, the above systems only consider the geometric information of the point cloud and ignore the intensity information, and the information is not fully utilized. In order to solve this problem, Tian et al. (2019) added the intensity difference of the points and the spatial Euclidean distance as constraints to the optimization solution of ICP, which greatly reduced the number of iterations of ICP, thus reducing the computational consumption of SLAM front-end registration [16]. Khan et al. (2016) proposed a LiDAR intensity information calibration method based on statistical methods and added the intensity difference constraint of the corresponding point in Hector-SLAM (Kohlbrecher et al., 2011) [17,18]. The test results showed that the positioning accuracy of the system had been improved. Similarly, Intensity SLAM (Wang et al., 2021) constructed a local intensity grid map and incorporated intensity information during scan-to-local map matching [19]. I-LOAM (Park et al., 2020) proposed an intensity difference weighting method for the feature point matching of LOAM, which assigned a higher weight to the corresponding points with similar intensity values [20].

As for tunnel environments, Li (2021) presented an intensity-enhanced laser SLAM approach based on LOAM [21]. The method first extracted geometric features and reflection intensity features from point clouds, then solved the degradation problem of geometric feature registration with the registration of intensity features and intensity maps. To investigate autonomous location and environment mapping of moving objects under conditions of dust and weak illumination in underground tunnels, Zhu (2019) proposed an improved EKF (Extended Kalman Filter) algorithm to perform fuzzy adaptive SLAM [22]. Prados et al. (2021) proposed a general SLAM model, which consisted of EKF, SMS (Scan Matching System), and GGO (Graph Generator and Optimizer) [23]. EKF can integrate a variety of odometry data, such as IMU odometry, wheel odometry, and visual odometry, then output the estimated pose for SMS. Finally, GGO optimized the pose and mapping results, which can realize positioning in the tunnel environment. Tardioli (2014) proposed a technique for localizing a robot in straight and smooth environments such as tunnels, mines, or pipes [24]. The method used exclusively a lidar sensor and a peculiar movement pattern, which allowed, by means of trivial trigonometric calculations, the computing of the longitudinal position with good precision, avoiding the necessity of feature matching. 

In summary, the existing laser SLAM can meet the matching and positioning requirements in most environments, but in the subway tunnel environment, although the geometric features in the tunnel are rich, the geometric features in the long and straight tunnels are degraded. The repeatability is high, meaning that it is very easy to mismatch during matching and positioning. Aiming at solving this problem, this paper studies the establishment and solution of the positioning model of laser SLAM in long straight tunnels, and optimizes the pose. It is mainly divided into the following parts:

- Feature extraction part. Firstly, we preprocess the tunnel point clouds. The principal axis direction of the point cloud is estimated by PCA (Principal Component Analysis) and corrected to the Y-axis (tunnel longitudinal direction). Secondly, the cross-section of the point cloud is obtained by using the straight-through filter and then fitted to a circle. Thirdly, we establish the polar coordinate system and extract the initial feature point set by comparing the polar diameter of each point. At last, a global geometric feature extraction model is constructed, and the features are extracted from the scan point cloud and map point cloud.
- Registration part. Aiming at the difference in point cloud density between scan point cloud and map point cloud, we propose a coarse-to-fine registration strategy. Firstly, we divide the coarse registration into two parts of rotation and translation to solve. The rotation matrix and translation matrix are obtained step by step by constructing the constrained registration between feature sets, completing the coarse positioning. On the basis of obtaining the initial pose, the point-to-plane ICP is used to complete the precise registration, and the final pose is solved, which solves the mismatch phenomenon in the subway tunnels.
- Pose optimization part. Aiming at the phenomenon that some scans have a large error in a certain direction, we define the scans with little registration error as keyframe. Then motion compensation will be applied to the scans with error uniform motion model. Finally, we optimize the poses according to the keyframe poses.

The remainder of this paper is structured as follows:

- Section 2 (Materials and Methods) details the experimental data acquisition using Innovusion lidar, the global geometric feature extraction model (including cross-section extraction, line/plane feature extraction, and convex hull feature extraction), the coarse-to-fine registration strategy (rotation-translation decomposition and Point-Plane ICP), and the keyframe-based pose optimization method.
- Section 3 (Results) validates the algorithm’s performance through feature extraction visualizations, registration accuracy metrics (RMSE), and comparative experiments with traditional ICP and PCA + ICP methods.
- Section 4 (Discussion and Conclusions) discusses the algorithm’s advantages in addressing geometric similarity challenges in tunnels, compares it with related works, and summarizes the quantitative improvements (3 cm accuracy, 0.7 s processing speed) and engineering applications.

## 2. Materials and Methods

### 2.1. Experimental Data

**Figure 1:**
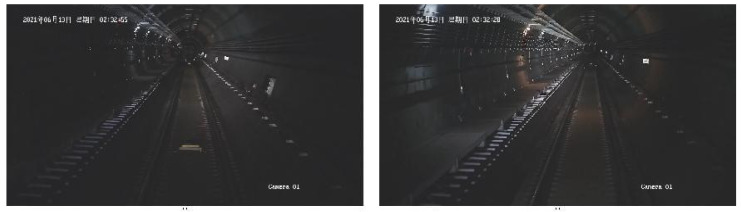
Shows the real subway tunnel environment.

The lighting conditions in real subway tunnels are not good, and there are similar geometric and texture features in the extension direction (Figure 1).

**Figure 2:**
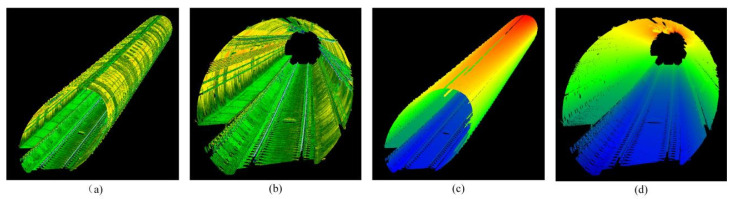
High-precision subway tunnel map point cloud. (**a**,**b**) Show the map point cloud rendered with reflection intensity. (**c**,**d**) Show the map point cloud rendered with Z-axis coordinates.

We used the Innovusion300 lidar for data collection. First, the lidar was mounted in front of the maintenance vehicle and rigidly connected to the high-precision IMU, and the two worked synchronously. The sensor coordinate system follows a right-handed convention: Y-axis along the tunnel’s longitudinal direction (vehicle forward), X-axis horizontal right, and Z-axis vertical up (zenith), as visualized by the Z-axis rendering in Figure 2c,d. Then we used the pose information of the IMU to stitch the single-frame scan point cloud to output the high-precision point cloud map of the tunnel, which can be used as the environment base map.

**Figure 3:**
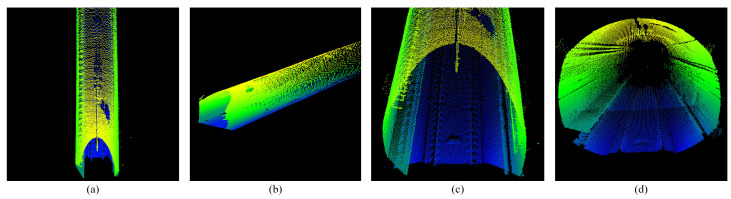
Single-scan point cloud of subway tunnel. All images are rendered with Z-axis coordinates. (**a**,**b**) Show the full view of the tunnel point cloud. (**c**,**d**) Show the tunnel point cloud detail.

Based on the construction of the environment map, we used the lidar to perform a secondary scan of the environment. This scan did not have IMU information, and the scan point cloud of each frame was reserved for matching and positioning (Figure 3).

### 2.2. Feature Extraction Model

Usually, the geometric features of 3D point cloud are mostly described by local single-point features such as corner points and plane points. In the environments with similar geometric structures, such as long straight tunnels, a large number of continuous repetitive features will appear. Therefore, we start with the point cloud as a whole and extract large-scale global features, including features such as lines, planes, and protrusions.

#### 2.2.1. Cross-Section Extraction

For the long straight tunnel environment, its shape can be approximated as a hollow cylinder arbitrarily distributed in space, and its cross-section is orthogonal to the central axis. The cross-section of the cylinder can be extracted by calculating the main axis of the cylinder. The specific calculation method is as follows:

1. Point cloud principal axis estimation

Equation (1) is used to compute the centroid as the basis for PCA. Alternative methods like the mean of maximum (MoM) were considered, but centroid calculation offers better stability for global feature extraction.

We first use the PCA to determine the main axis of the point cloud. For a set of point clouds $\left\{p_{1},p_{2}\ldots p_{N}\right\}$, we calculate its center of gravity $\bar{p}$ by Equation (1):

(1) $$\bar{p}=\frac{1}{N}\sum_{i=1}^{N}p_{i}$$

Then we construct its covariance matrix R by Equation (2):

(2) $$R=\frac{1}{N}\sum_{i=1}^{N}\left(p_{i}-\bar{p}\right)$$

Finally, we perform SVD (Singular Value Decomposition) on the matrix R to obtain three eigenvalues $\lambda_{1},\lambda_{2},\lambda_{3}$ (as a vector) and the corresponding eigenvectors $v_{1},v_{2},v_{3}$, among which the eigenvector $v_{i}$ corresponding to the largest eigenvalue $\lambda_{i}$ is the main direction of the point cloud.

2. Tunnel coordinate system establishment

**Figure 4:**
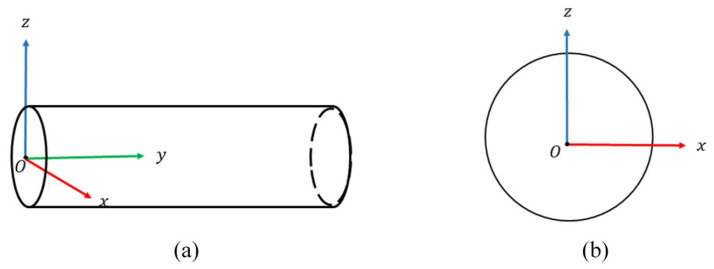
Schematic diagram of the tunnel coordinate system. (**a**) Front view. (**b**) Left view.

In order to facilitate the calculation, we transform the estimated main direction v of the point cloud by rotation, so that it coincides with the Y-axis. At this time, the tunnel extends along the axis direction; the zenith direction of the tunnel is the Z-axis. The plane formed by the two axes is orthogonal to the X-axis. The XOZ plane corresponds to the cross-section of the point cloud (Figure 4).

3. Cross-section extraction.

**Figure 5:**
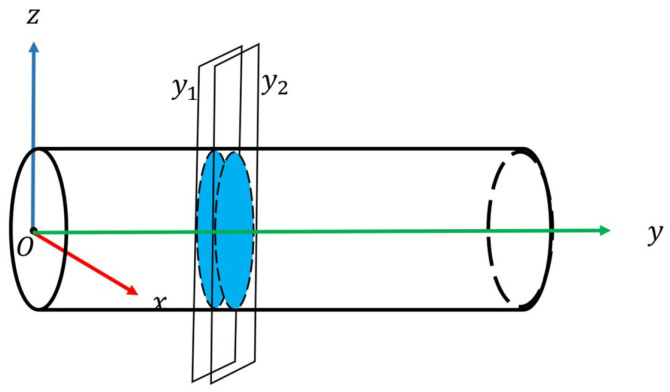
Extract cross-section of the point cloud by pass-through filtering on the Y-axis.

**Figure 6:**
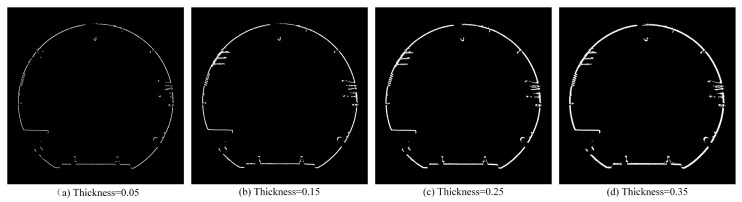
Front view of cross-sections of point clouds with different thicknesses.

**Figure 7:**
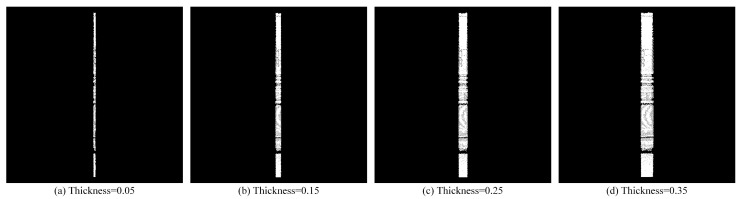
Left view of cross-sections of point clouds with different thicknesses.

Pass-through filter is a filtering method that can filter out points whose values in a specified dimension direction are not within a given range. On the basis of the constructed tunnel coordinate system, the cross-section of the point cloud can be extracted directly using the pass-through filter in the Y-axis. According to the density distribution of the point cloud on the Y-axis, select the appropriate value range $\left[y_{1},y_{2}\right]$ to extract the cross-section of the point cloud. The interval [y1, y2] is set to 5 cm (thickness) through experiments, balancing feature integrity and computational efficiency (Figure 5, Figure 6 and Figure 7).

#### 2.2.2. Initial Feature Point Set Extraction

After extracting the cross-section of the point cloud, in order to quickly extract global features, we perform a series of processing on the cross-section to obtain some feature points as the initial feature point set.

1. Fit circular point cloud

Due to the partial aggregation features inside the point cloud, its cross-section is not an absolutely regular circle. There are protrusions or depressions in some areas, and the overall trend is circular. Therefore, we can use the Random Sample Consensus (RANSAC) algorithm to fit the cross-section to a circle. The circle equation can be described as Equation (3):

(3) $${\left(x-x_{0}\right)}^{2}+{\left(z-z_{0}\right)}^{2}=r^{2}$$

where $O\left(x_{0},y_{0},z_{0}\right)$ is the center of the circle and r denotes the fitted circle radius.

2. Rearrange the numbers of points

After the cross-section is extracted, the numbers of each point are not arranged in order, which is not conducive to calculating features through neighbors. Therefore, we first convert the cross-section point cloud coordinates into polar coordinates. The polar axis is the vector composed of the center and the point with the largest coordinate in the point cloud by Equation (4):

(4) $$n=\left(x-x_{0},y-y_{0},z_{max}-z_{0}\right)$$

Then the polar coordinates of each point can be calculated by Equation (5):

(5) $$\theta_{i}=a\;cos(n*n_{i}/R^{2})$$

where $n_{i}$ is the vector formed by each point and the center of the circle. After converting to polar coordinates, we arrange the points according to the angular coordinate θ from small to large, and renumber them in turn.

3. Extract the initial feature point set

The simplified cross-section of the tunnel point cloud can be seen as composed of smooth surfaces, track lines, platform planes, and some noisy point clouds. According to the actual spatial characteristics of the tunnel, the cross-section point cloud can be divided into three types: surface point, plane point, and straight-line point, which can be extracted separately through the relationship between polar diameter r and radius R.

**Figure 8:**
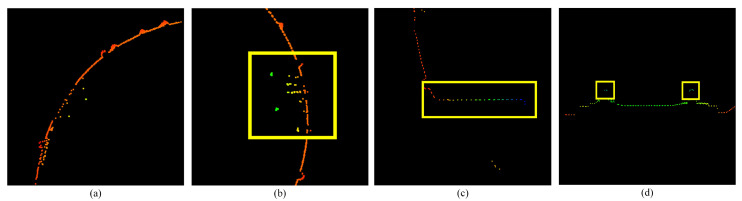
Shows the points in different regions. (**a**) Smooth points. (**b**) Noise points. (**c**) Plane points. (**d**) Straight-line points.

**Figure 9:**
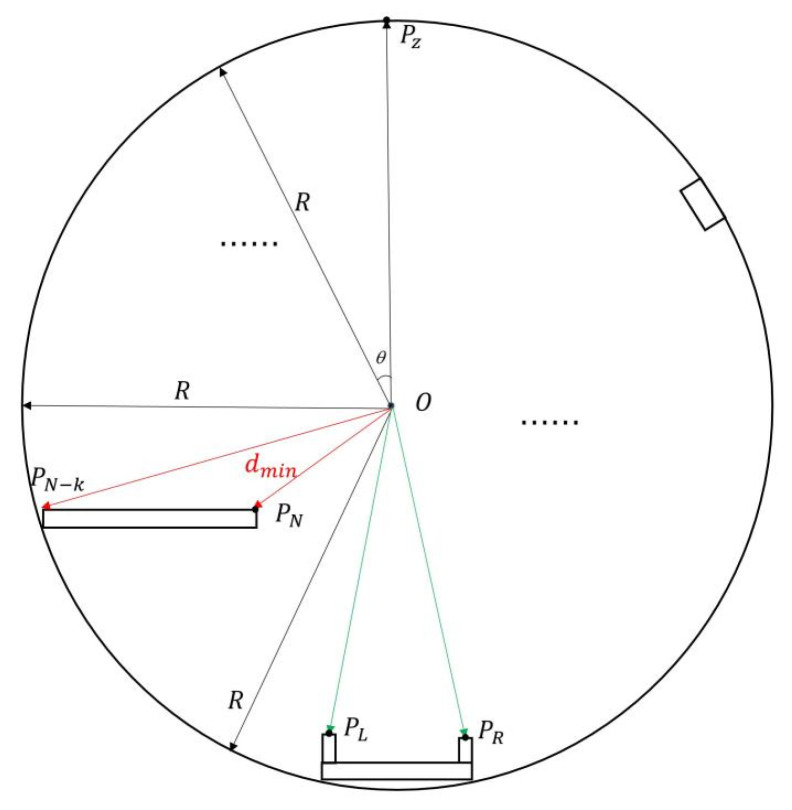
Describe the distribution of the three types of feature points in the point cloud of the tunnel section, and the relationship between the corresponding polar diameter r and radius R.

- If r≈R (within ± 0.05 m, 2% of tunnel radius), it is considered that the point is on or near the fitted circle and can be classified as a surface point.
- If r≪R, and $r=min\{r_{i},\;i=1,2,3\ldots n\}$, the point can be considered as the starting point of the plane. Its point number is N, and the point is denoted as $p_{N}$. After a small threshold ε is given, k points are taken backward and all points satisfying $\left|r_{N+i}-R\right|>\varepsilon$ $\left(i=1,2\ldots k\right)$ are recorded as plane point set S (Figure 8c).
- Among the remaining points, in the same way as above, two points $P_{L}$ and $P_{R}$ (left/right track lines) with the smallest r are extracted as straight-line points and recorded as straight-line point set L (Figure 8d and Figure 9).

#### 2.2.3. Line and Plan Feature Extraction

By processing the two sets of cross-sections of point clouds, we can obtain the initial feature point set S of the plane, which corresponds to the tunnel platform plane; the initial feature point sets $L_{1}$ and $L_{2}$ of the two straight lines correspond to the left and right track lines, respectively. On this basis, the mathematical models are calculated, respectively, and the global line and plane features are extracted.

1. Line feature extraction

**Figure 10:**
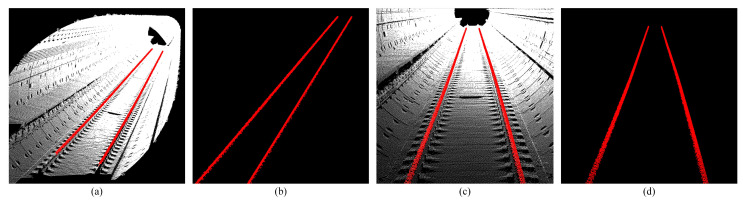
Shows the extraction results of straight-line features. (**a**,**b**) Show side view track line extraction. (**c**,**d**) Show front view track line extraction.

Two points are randomly selected from the initial feature set $L_{1}$ of the line (e.g., Line1: direction vector (0.999, −0.004, 0.019)) to calculate the equation of the line composed of the two points. Points within ε = 0.03 m distance to the line are classified as line features (Figure 10). The equation is in the form of Equation (6):

(6) $$\frac{x-x_{0}}{m}=\frac{y-y_{0}}{n}=\frac{z-z_{0}}{p}$$

where $\left(x_{0},y_{0},z_{0}\right)$ is a point on the line, and $\left(m,n,p\right)$ is the direction vector of the line.

Then calculate the distance $d_{i}$ from each point in the global point cloud to the line. Given a tolerance threshold ε, all points satisfying $d_{i}\leq \varepsilon$ can be considered as line points. Perform the same processing on another set of line points $L_{2}$, thus completing the line feature extraction.

2. Plane feature extraction

**Figure 11:**
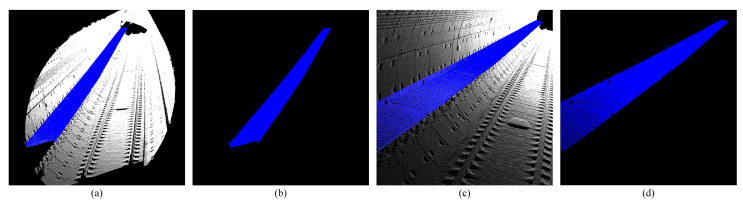
Shows the extraction results of plane features. (**a**,**b**) Show side view track plane extraction. (**c**,**d**) Show front view track plane extraction.

Three points are randomly selected from the initial feature set S of the plane to calculate the equation of the plane composed of the three points (e.g., plane normal vector (−0.019, 0.017, 0.999)). Points within ε = 0.02 m distance to the plane are plane features (Figure 11). The equation is in the form of Equation (7):

(7) $$A\left(x-x0\right)+B\left(y-y0\right)+C\left(z-z0\right)=0$$

where $\left(x_{0},y_{0},z_{0}\right)$ is a point on the plane and $\left(A,B,C\right)$ is the normal vector of the plane.

Then calculate the distance $d_{i}$ from each point in the global point cloud to the plane. Given a tolerance threshold ε, all points satisfying $d_{i}\leq \varepsilon$ can be considered as plane points, thus completing the surface feature extraction.

#### 2.2.4. Convex Hull Feature Extraction

**Figure 12:**
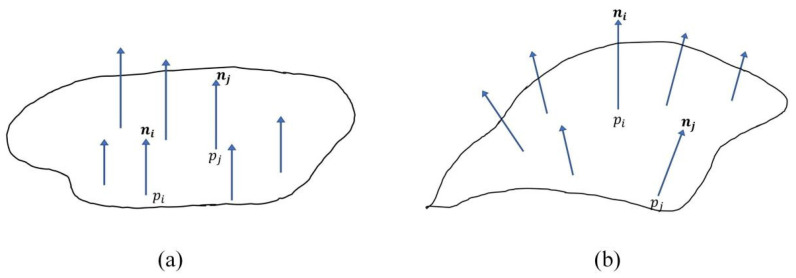
Describes the change of the normal vector of the points in different regions. (**a**) The normal vectors of the points in the flat region are approximately parallel. (**b**) There is a large angle between the normal vectors of the points in the undulating area.

The normal vector of any point in a 3D point cloud is perpendicular to the tangent plane where the point lies. For a plane in space, its normal vector is perpendicular to the plane, so there are countless normal vectors, which are parallel to each other. For the normal vector of each point on the surface, there will be big differences due to the degree of curvature of the surface and the position of the point on the surface (Figure 12).

The change of the normal vectors of the points in the local neighbors is an important geometric feature of the surface, which can well reflect the bending degree of the local surface formed by the point and its neighbors. Therefore, we define sharp bulges on smooth surfaces as convex hulls. There will be a large angle between the normal vectors corresponding to this area.

**Figure 13:**
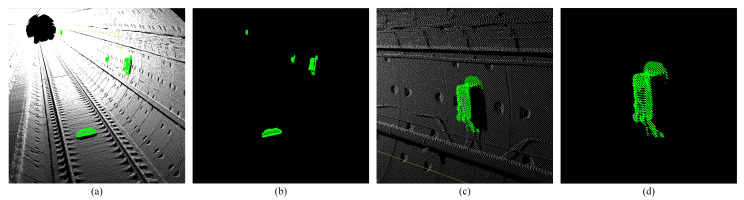
Shows the extraction results of convex hull features. (**a**,**b**) Show the convex hull along the middle line of the track. (**c**,**d**) Show the convex hull along the middle line of the right tunnel wall.

In order to describe the difference between the convex hull and the surrounding smooth region, we give the definition of the rate of change of the normal vectors at a point p by Equation (8):

(8) $$rate_{\theta }=\sum_{i=1}^{N}\theta_{i}/N$$

where $\theta_{i}$ is the angle formed by the normal vector of each point $p_{i}$ in the neighborhood and the normal vector of the point p, and N is the number of neighbour points. The steps to extract the convex hull feature by the normal vector change rate are as follows:

- For a point p, given the neighbour radius r, obtain the neighbour point set S of the point p through KD tree query. Neighbor radius r = 0.2 m (covering typical protrusions like brackets) is used to query neighbors via KD tree.
- Estimate the normal vector n of point p and the normal vector $n_{i}$ of point $p_{i}\left(i\leq N,p_{i}\in S\right)$ by means of local surface fitting, and then calculate the angle $\theta_{i}$ between them by Equation (9):
  (9) $$\theta_{i}=arccos\left(n\cdot n_{i}/\left|\left|n\right|\left|n_{i}\right|\right|\right)$$
- Calculate the normal vector change rate $rate_{\theta }$ of point p. Given a threshold ε, points whose $rate_{\theta }$ are greater than the threshold can be considered to be the convex hull features, thus completing the extraction. Threshold ε = 0.1 rad for normal vector change rate (Equation (8)) identifies convex hulls. Only features on the right tunnel wall and track midline are extracted to reduce noise from pipes and improve registration efficiency (Figure 13).

Due to the huge number of global point clouds, various parts such as wires and brackets in the real environment will also be considered as convex hull features. In order to reduce the influence of the noise point cloud and improve the efficiency of the program, we only extract the convex hull features appearing on the tunnel wall on the right side and the midline of the track for registration.

### 2.3. Coarse-to-Fine Registration Strategy

After completing the feature extraction, registration needs to be performed to solve the relative transformation between the scan and the map point clouds. Compared with the single-scan point cloud, the number of map point clouds is very large, and there may be a large number of similar geometric texture structures in the environment. Conventional coarse registration methods may provide a large deviation of the initial pose, which will lead to a local optimum in the fine registration stage. Therefore, we start with the distribution of geometric feature sets in space and complete the coarse registration by dividing it into rotation and translation. Then the obtained initial pose is used for the fine registration of large-scale similar scenes, and finally we complete the matching and positioning.

#### 2.3.1. Step-by-Step Coarse Registration

In order to complete the rough registration quickly and accurately, we divide the process of solving the transformation matrix into two independent parts: rotation and translation. The rotation matrix is solved by the registration between the line features, and the translation matrix is solved jointly by the registration between the plane features and the registration between the convex hull features.

**Figure 14:**
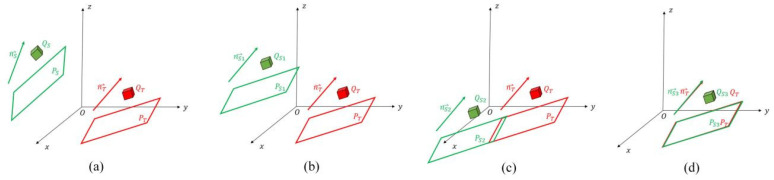
Describes the process of coarse registration. (**a**) Shows the initial positions of the feature sets in the scan point cloud and the map point cloud. (**b**) After the rotation transformation, the two sets of feature point clouds are in parallel. (**c**) After the first translation transformation, the positions of the two sets of feature point clouds. (**d**) After the second translation transformation, the positions of the two sets of feature point clouds.

There are existing source scan point cloud S, target map point cloud T, line feature sets $L_{S}$ of the S and $L_{T}$ of the T, plane feature sets $P_{S}$ and $P_{T}$, convex hull feature sets $Q_{S}$ and $Q_{T}$, all of which are respectively obtained from the feature extraction in Section 2.2. And according to the fitted line and plane equation in Section 2.2.3, the direction vectors of the straight lines $n_{S}$ and $n_{T}$ and the plane normal vector n can be obtained, respectively. Figure 14a abstractly represents the positional relationship between them.

1. Solve the rotation transformation by line features

We first transform the line feature direction vector $n_{S}$ of the scan to be parallel to the line feature direction vector $n_{T}$ of the map. The rotation can be represented by a rotation axis N perpendicular to the plane formed by the two vectors and a rotation angle θ in the plane. These two parameters can be solved by Equation (10):

(10) $$\left\{\begin{array}{c} N=n_{S}\times n_{T}\\ \theta =arccos\left(\left(n_{S}\cdot n_{T}\right)/\left|\left|n_{S}\right|\left|n_{T}\right|\right|\right) \end{array}\right.$$

The rotation matrix R corresponding to the rotation vector θN can be calculated by Rodrigues’s Formula, Equation (11):

(11) $$R=cos\left(\theta \right)I+(1-cos(\theta ))NN^{T}+sin(\theta )Skew(N)$$

where $N=\left(N_{x},N_{y},N_{z}\right),\;\;Skew\left(N\right)=\left[\begin{array}{ccc} 0 & -N_{z} & N_{y}\\ N_{z} & 0 & -N_{x}\\ -N_{y} & N_{x} & 0 \end{array}\right]$.

2. Solve the translation transformation by line features

Apply the rotation transformation to the plane feature $P_{s}$ of scan and the convex hull feature $Q_{s}$ of scan, so that we can obtain the new plane feature $P_{s1}$ and convex hull feature $Q_{s1}$ after the rotation by Equation (12), as shown in Figure 14b.

(12) $$\left\{\begin{array}{c} P_{S1}=R\cdot P_{S}\\ Q_{S1}=R\cdot Q_{S} \end{array}\right.$$

At this time, the scan point cloud has completed the registration of the rotation part, and the two sets of point clouds are in parallel. However, there are still displacement deviations in the spatial position. Therefore, it is necessary to translate the scan point cloud to align it to the approximate position of the map point cloud.

Take any point $p_{1}$ in $P_{S1}$ and point $p_{2}$ in $P_{T}$, and calculate the distance d between the two points and the vector l formed by the two points.

Calculate the angle θ between the vector l and the normal vector n of the plane $P_{T}$ of the map by Equation (9), so as to obtain the vertical distance h between the two planes by Equation (13).

(13) $$h=d*cos\left(\theta \right)$$

If the normal vector n is the normalized vector $\left(e_{x},e_{y},e_{z}\right)$, then the translation matrix can be solved by Equation (14).

(14) $$T_{1}=h\cdot n$$

Otherwise, normalize the normal vector before calculating the translation matrix $T_{1}$.

Apply the translation transformation to the first-transformed convex hull feature $Q_{S1}$ to obtain the second-transformed convex hull feature set $Q_{S2}$, as shown in Figure 14c.

Repeat the above, only replacing the points $p_{1}$ and $p_{2}$ with the geometric centers of $Q_{S2}$ and $Q_{T},$ respectively, and replacing the normal vector n with the straight-line direction vector. In the same way, the secondary translation matrix $T_{2}$ can be obtained. At this time, the scan point cloud has been transformed to the approximate position of the map point cloud, as shown in Figure 14d.

After the above transformation steps, the rotation matrix R and the two translation matrices $T_{1}$ and $T_{2}$ are obtained in sequence. Finally, the coarse transformation of the scan point cloud to the map point cloud can be achieved by Equation (15) as follows:

(15) $$S_{C}=R\cdot S+\left(T_{1}+T_{2}\right)$$

where $S_{C}$ is the scan point cloud after coarse registration.

#### 2.3.2. Point-Plane Iterative Closest Points Registration

After completing the coarse registration, the scan point cloud has been transformed to the position near the map point cloud, which has a good initial pose, and a more accurate transformation matrix needs to be solved. The traditional ICP algorithm uses the Euclidean distance to retrieve the closest point from the target point cloud. The optimal transformation matrix is iteratively solved by minimizing the distance between the corresponding points as the registration criterion. However, most of the area in the tunnel point cloud is approximately a smooth surface, so constructing the distance constraint between point and the tangent plane of the corresponding point can better reflect the spatial structure of the point cloud and resist the influence of wrong corresponding point pairs. The registration method needs to provide the normal vector of each point, and the vectors have been calculated in the convex hull extraction part, which can be directly applied here to further speed up the calculation efficiency.

**Figure 15:**
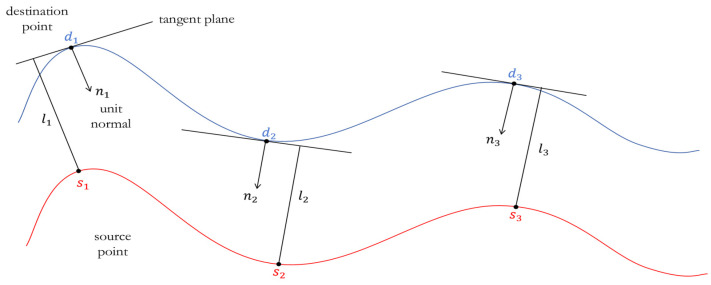
Describes how to calculate the distance from the scan point to the tangent plane of the corresponding map point.

The precise registration method of Point-Plane ICP uses the square of the distance from the scan point to the tangent plane of the corresponding map point as the loss function and minimizes the loss function to obtain the optimal transformation matrix (Figure 15).

The mathematical expression of the loss function is as in Equation (16), as follows:

(16) $$R_{opt}={argmin}_{R}\sum_{i}{\left(\left(R\cdot s_{i}-d_{i}\right)\cdot n_{i}\right)}^{2}$$

where $s_{i}$ is the scan point, $d_{i}$ is the corresponding map point, $n_{i}$ is the normal vector of $d_{i}$. Iteratively calculate the transformation matrix until the distance between the point clouds is less than a certain threshold or reaches the pre-set maximum number of iterations. At this time, the fine registration of the scan point cloud to the map point cloud is completed, and the final fine registration transformation matrix is $R_{opt}$.

### 2.4. Pose Optimization

Since the convex hull features are distributed discontinuously in the tunnel, some scans may fail to extract the convex hull feature during the scanning process, which results in the loss of constraints in the extension direction of the tunnel. Therefore, the second translation matrix cannot be solved, and the pose estimation result is inaccurate.

To solve this problem, we propose a pose optimization method based on keyframes and motion compensation.

#### 2.4.1. Select Keyframe

We first give the definition of the keyframe. Keyframes are defined as scans with a >70% convex hull matching rate and <10 ICP iterations, assumed accurate for local optimization. Global consistency is ensured by subsequent pose graph optimization. The scan point cloud that has extracted obvious convex hull feature and completed the accurate registration is regarded as the keyframe, and its pose result can be considered to be accurate.

**Figure 16:**
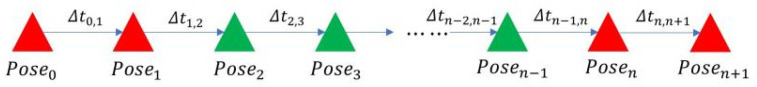
Shows the chronological order of keyframes and non-keyframes. Red triangles represent keyframes and green triangles represent non-keyframes.

In the actual scanning process, the relationship between keyframes and non-keyframes is shown in Figure 16.

After registration, the pose of each frame of point cloud at the scan time is calculated as $Pose_{i}=\left[x_{i},y_{i},z_{i},roll_{i},pitch_{i},yaw_{i}\right]$. For keyframes, $Pose_{i}$ is considered to be accurate and error-free. For other frames, errors occur only because of the lack of convex hull feature constraints on the Y-axis. The X and Z positions and attitude angles can also be considered accurate, so we only need to correct the Y-axis position.

#### 2.4.2. Motion Compensation Model

Since the scanning frequency of lidar is stable at 50 Hz, and the scanning interval between frames is about 0.02 s, we believe that the moving speed is approximately constant in a very short period of time, which is a uniform motion model. So, for keyframes 0 and 1 in Figure 16, the running speed can be estimated from the pose change by Equation (17).

(17) $$v_{y}=\left(y_{1}-y_{0}\right)/\Delta t_{0,1}$$

For subsequent non-keyframes, use the uniform motion model to correct their Y-axis coordinates $y_{i}$ by Equation (18).

(18) $$y_{i}=y_{1}+\sum \Delta t_{i,i+1}*v_{y}$$

where $\Delta t_{i,i+1}$ is the time interval between the $i_{th}$ frame and the ${i+1}_{th}$ frame.

#### 2.4.3. Optimized Adjustment

Continuously estimate the non-keyframe poses for the uniform motion model until the next keyframe is scanned. At this time, the pose of the keyframe obtained by the estimated model is recorded as $Pose_{n}=\left[x_{i},y_{i},z_{i},roll_{i},pitch_{i},yaw_{i}\right]$.

Since the first and $n_{th}$ frames are keyframes, their poses $Pose_{1}$ and $Pose_{n}$ are accurate. As for the second frame to the ${n-1}_{th}$ frame, there will be errors in their poses, and error propagates cumulatively to the $n_{th}$ frame. So, we first take $\hat{Pose_{n}}$ as the estimated value and $Pose_{n}$ as the true value, then calculate the pose error δ by Equation (19) as follows:

(19) $$\delta =Pose_{n}-\hat{Pos}e_{n}$$

Then we define the weight model according to the time interval between frames. The weight of the $i_{th}$ frame can be calculated by Equation (20).

(20) $$w_{i}=\Delta t_{i,i+1}/t$$

where t is the total time from the first frame to the $n_{th}$ frame. Finally, the $i_{th}$ corrected pose $\bar{Pose_{i}}$ can be calculated by Equation (21).

(21) $$\bar{Pose_{i}}=Pose_{i}+w_{i}\cdot \delta$$

## 3. Results

In this section, real subway tunnel laser point cloud data are used to verify the effectiveness of our algorithm by C++ in Visual studio 2019 as assisted by The Point Cloud Library 1.8.0 (PCL 1.8.0) programming.

### 3.1. Feature Extraction Results

#### 3.1.1. Cross-Section Extraction Results

According to the cross-section extraction model in Section 2.2.1, we can obtain cross-sections of point clouds with different thicknesses by setting different pass-through filtering intervals.

A 5 cm thickness is validated as optimal: thinner sections (e.g., 3 cm) lose features (Figure 6a), while thicker sections (e.g., 10 cm) increase noise (Figure 7b).

When the thickness of the section is too small, some features may be missing on the section. When the thickness is too large, the local point cloud is denser, which increases the amount of calculation. Therefore, after many experiments, there is a better extraction effect when the section thickness is set to 5 cm.

#### 3.1.2. Initial Feature Point Set Extraction Results

**Figure 17:**
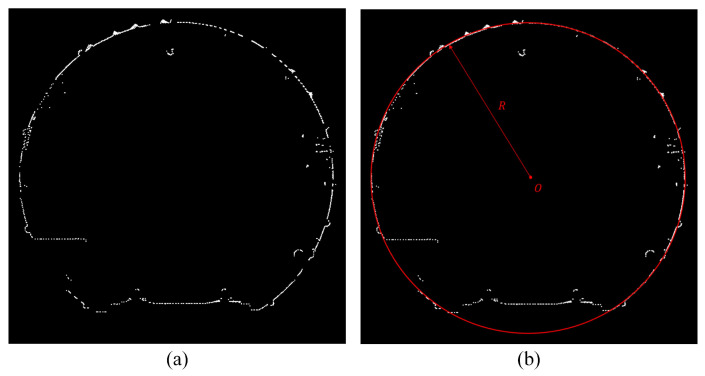

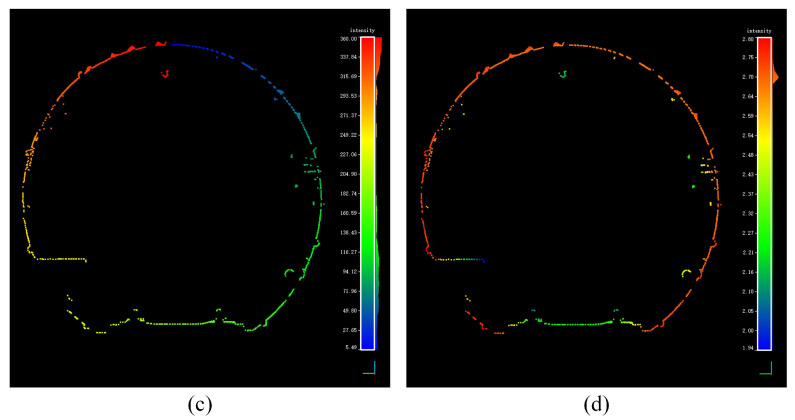
Processing steps for cross-section point cloud. (**a**) Shows the original 5 cm-thick cross-section point cloud. (**b**) Shows the fitted circle of cross-section point cloud. (**c**) Shows the cross-section point cloud after rearranging, and it is rendered by the number. (**d**) Shows the cross-section point cloud which is rendered by the distance from each point on the section to the center of the fitted circle.

Fit the obtained cross-section of point cloud to a circle, then we can obtain the model equation. On this basis, we arrange the point number by the model in Section 2.2.1. Then we calculate the polar coordinates of each point on the cross-section (Figure 17).

The entire cross-section consists of smooth points, noise points, plane points, and straight-line points. The details of each part are shown in Figure 8.

We arrange the polar diameter in the order of the point number. The straight-line points and plane points can be extracted through the extraction model in Section 2.2.2.

#### 3.1.3. Line and Plane Feature Extraction Results

On the basis of obtaining the initial plane feature point set and the initial line feature point set, the line equation and the plane equation parameters can be solved by the extraction model in Section 2.2.3.

The equations of the two lines and the plane are as follows:

$$Line1:\frac{\left(x-27.362\right)}{0.999}=\frac{\left(y-0.499\right)}{-0.004}=\frac{\left(z+3.591\right)}{0.019}$$

$$Line2:\frac{\left(x-26.990\right)}{0.999}=\frac{\left(y+1.015\right)}{-0.004}=\frac{\left(z+3.578\right)}{0.019}$$

Plane:−0.019x+0.017y+0.999z+3.193=0

Then we can extract the global line and plane features of the point cloud.

**Figure 18:**
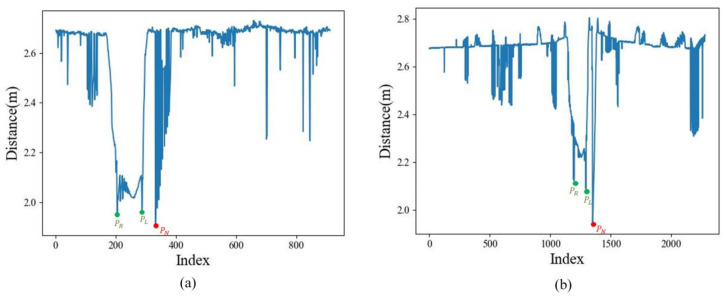
Shows the variation of polar diameter of each point in the cross-section point cloud, where $p_{N}$ is the plane point, $p_{L}$ and $p_{R}$ are the line points. (**a**) Cross-section of map point cloud. (**b**) Cross-section of scan point cloud.

It can be seen that the track line and platform plane in the tunnel are completely extracted. Manual annotation of 100 frames shows 92% recall for plane points, 95% precision for line points, and 88% F1-score for convex hulls (Figure 8 and Figure 18).

#### 3.1.4. Convex Hull Feature Extraction Results

**Figure 19:**
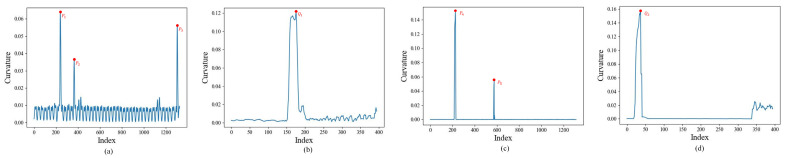
Shows the trend of the rate changing along the line. (**a**) Shows the trend along the middle line of the track in the map point cloud. (**b**) Shows the trend along the middle line of the right tunnel wall in the map point cloud. (**c**) Shows the trend along the middle line of the track in the scan point cloud. (**d**) Shows the trend along the middle line of the right tunnel wall in the scan point cloud.

We first calculate the rate of change of normal vectors for the point cloud along the middle line of the track and the middle line of the right tunnel wall through Equation (8) in Section 2.2.4. Then we can obtain the trend of the rate changing along the line(Figure 19).

It can be clearly seen that the normal vector change rates fluctuate around 0 in most areas, indicating that this part of the area is smooth without obvious fluctuations. However, in the part marked by the red point in the figure, the change rate of the normal vector has increased sharply, indicating that the convex hull feature appears in this part. By extracting this kind of mutation point and its neighboring points, the convex hull extraction is completed.

**Figure 20:**
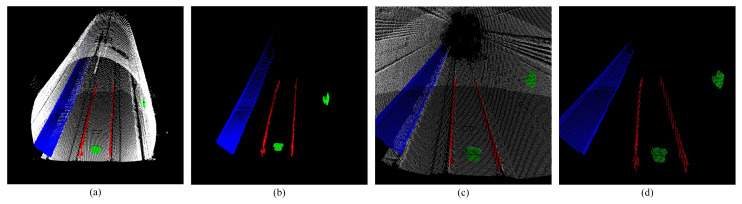
Shows the extraction results of three types of features, where the red represents the line features, the blue represents the plane features, and the green represents the convex hull features. (**a**,**b**) Show front view extraction results of three types of features. (**c**,**d**) Show side view extraction results of three types of features.

Similarly, by processing a single scan of the point cloud, the extraction results of three types of feature sets of straight line, plane, and convex hull can also be obtained (Figure 20).

### 3.2. Registration Result

#### 3.2.1. Coarse Registration Results

**Figure 21:**
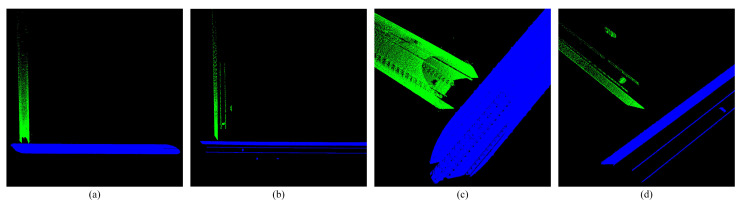
Shows the spatial initial positions of the two sets of point cloud. (**a**,**c**) Show the positions of point clouds. (**b**,**d**) Show the positions of features.

After completing the feature extraction, the spatial initial positions of single-scan point cloud and map point cloud are shown in Figure 21.

First, the line features of the two sets of point clouds are rotated to parallel according to their respective direction vectors. The line feature direction vectors of the scan point cloud and map point cloud fitted in Section 3.1.3 are $n_{S}$ and $n_{T}$, respectively.

$$n_{S}=\left(0.019,-0.999,-0.014\right)\;n_{T}=\left(0.999,-0.004,\;0.019\right)$$

The rotation axis N and the rotation angle θ can be obtained by Equation (10) when $n_{S}$ is rotated to be parallel to $n_{T}$.

$$N=\left(-0.019,\;-0.033\;,0.998\right)\;\theta =88{}^{\circ}41^{\prime }55^{\prime \prime }$$

Then, the transformation matrix R is obtained by the Rodrigues formula.

$$R=\left[\begin{array}{ccc} -0.0205928 & 0.9997690 & -0.0062183\\ -0.9992550 & -0.0207846 & -0.0325323\\ -0.0326541 & 0.0055437 & 0.9994510 \end{array}\right]$$

**Figure 22:**
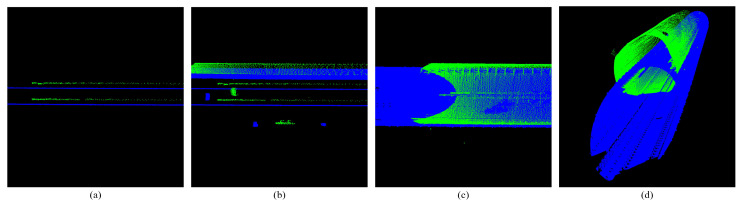
Shows the result after the rotation transformation of coarse registration. (**a**) Shows the line features in parallel of the two sets of point clouds. (**b**) Shows all the features in parallel of the two sets of point clouds. (**c**,**d**) Show the two parallel sets of point clouds.

After the rotation matrix is applied to the frame point cloud, the first step of coarse registration is completed (Figure 22).

**Figure 23:**
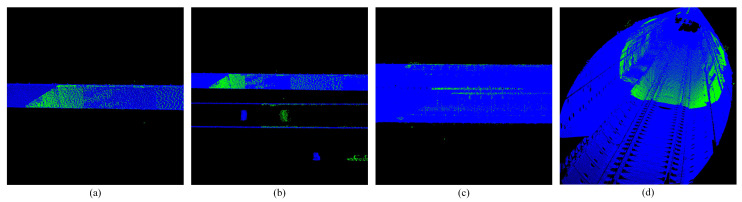
Shows the result after the first translation transformation of coarse registration. (**a**) Shows the coincident plane features of the two sets of point clouds. (**b**) Shows the coincident line and plane features of the two sets of point clouds. (**c**,**d**) Show the two coincident sets of point clouds.

Then apply the above rotation transformation to the plane features of the scan point cloud and calculate the vertical distance between the transformed plane and the map plane (Figure 23). Translate the plane of scan point cloud along the plane normal vector direction and the first translation matrix $T_{1}$ can be solved.

$$T_{1}={\left[\begin{array}{ccc} 0.0245 & -0.2679 & -1.9461 \end{array}\right]}^{T}$$

**Figure 24:**
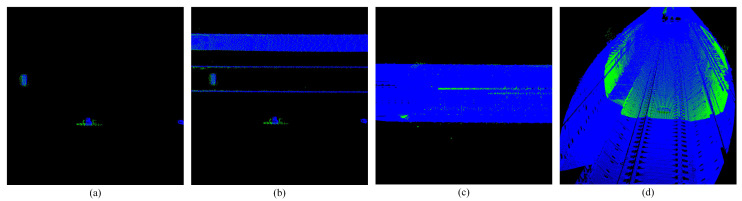
Shows the result after the second translation transformation of coarse registration. (**a**) Shows the coincident convex hull features of the two sets of point clouds. (**b**) Shows all the coincident features of the two sets of point clouds. (**c**,**d**) Show the two coincident sets of point clouds.

Finally, apply R and $T_{1}$ to the convex hull features of the scan point cloud, calculate the distance between the center of the convex hull after the transformation and the center of the convex hull in the map (Figure 24). Translate along the direction vector of the track line to obtain the second translation matrix $T_{2}$.

$$T_{2}={\left[\begin{array}{ccc} -2.6632 & 0.0098 & -0.0506 \end{array}\right]}^{T}$$

The final coarse registration transformation matrix is $R_{coarse}$.

$$R_{coarse}=\left[\begin{array}{cccc} -0.0205928 & 0.9997690 & -0.0062183 & -2.6387\\ -0.9992550 & -0.0207846 & -0.0325323 & -0.2581\\ -0.0326541 & 0.0055437 & 0.9994510 & -1.9967\\ 0 & 0 & 0 & 1 \end{array}\right]$$

#### 3.2.2. Fine Registration Results

On the basis of completing the rough registration, a fine registration is performed on the scan point cloud, and the fine registration matrix is $R_{Fine}$.

$$R_{Fine}=\left[\begin{array}{cccc} 1 & 0 & 0 & 0.021\\ 0 & 1 & 0 & -0.020\\ 0 & 0 & 1 & 0.012\\ 0 & 0 & 0 & 1 \end{array}\right]$$

**Figure 25:**
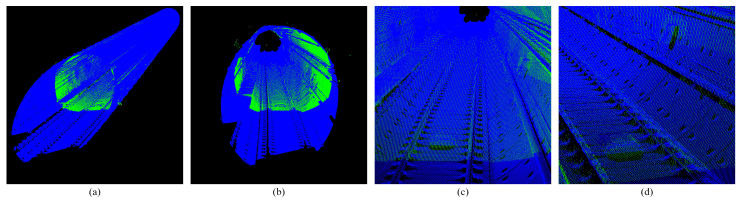
Shows the result after fine registration. (**a**,**b**) Show the full view after fine registration. (**c**,**d**) Show the detail after fine registration.

It can be seen that the rotation component in the fine registration matrix is an identity matrix. After the constrained registration between feature sets in the coarse registration process, the pose angle in the pose of the scan point cloud has been determined. The fine registration only effects a slight translation along the three axes, which can be verified by the small amount of the translation component of the fine registration matrix (Figure 25).

#### 3.2.3. Pose Optimization Results

**Figure 26:**
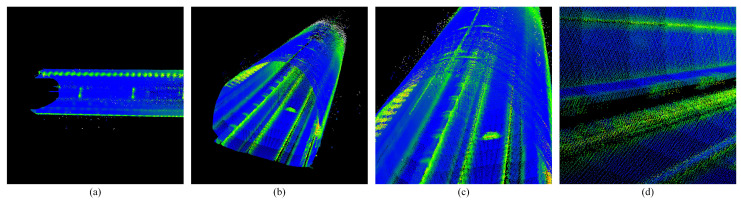
(**a**,**b**) Show the mapping results before optimization. (**c**,**d**) Show the drift in details.

We use the algorithm in this paper to continuously process multiple scans of point clouds, solve the pose of each scan of point cloud, then splice them to build a map. Since part of the mapping details drift can be visually displayed through the displacement of the convex hull, the mapping results in this section are all rendered with the point cloud normal vector change rate (Figure 26).

**Figure 27:**
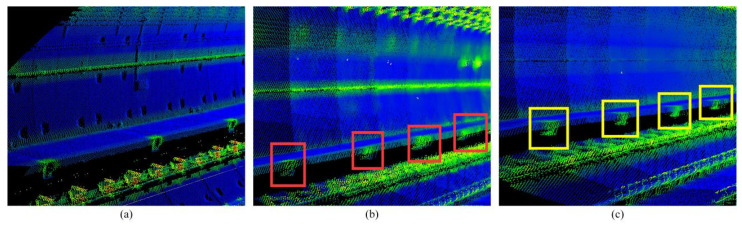
Shows the comparison of mapping details before and after optimization. (**a**) Shows the details in the original map point cloud. (**b**) Shows the drift details in the mapping result before optimization. (**c**) Shows the corrected drift details in the mapping result after optimization.

Since some scans are not constrained along the extension direction of the tunnel during registration, there will be mismatches in the extension direction. The registration error is mainly concentrated in this direction, resulting in drift in some positions. The normal position of this part in the map point cloud is shown in Figure 27a, and the drift error during the mapping process is shown in Figure 27b. After the keyframe-based pose optimization method in this paper, the error correction is shown in Figure 27c.

**Figure 28:**
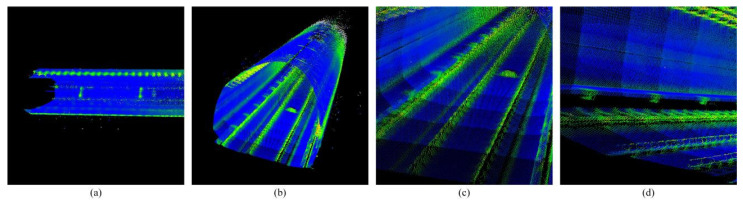
Shows the final optimized mapping results. (**a**,**b**) Show the full view after final optimized mapping. (**c**,**d**) Show the detail after final optimized mapping.

From Figure 28, it can be seen that there is basically no error in the positions of the X and Z axes before and after optimization because in the rough registration process, the registration between the line features constrains the error in the X-axis direction, and the registration between the plane features constrains the error in the Z-axis direction, and the errors of the two axes are controlled at smaller magnitude. However, the point clouds of some scans before optimization lack the registration constraint of the convex hull features along the Y-axis direction of the tunnel extension, resulting in a large registration error. After optimization, drift correction is performed on the originally constructed point cloud map, so the registration error of the Y-axis is significantly reduced. The drift phenomenon of some scans is solved, the pose results are more accurate, and the details in the map are also better represented, achieving a better registration effect. It is of great significance for matching and positioning in the subway tunnels with similar structure.

#### 3.2.4. Comparative Experiment

We use the Root Mean Square Error (RMSE) as the similarity measure to evaluate the registration accuracy between the scan point cloud and the map point cloud and evaluate the registration efficiency by the program running time.

(22) $$RMSE=\sqrt{\frac{1}{n}\sum_{i=1}^{n}{\left(X_{i}-{\hat{X}}_{i}\right)}^{2}}$$

where $X_{i}$ and ${\hat{X}}_{i}$ represent the corresponding point pairs in the source point cloud and the target point cloud, respectively, and n is the number of corresponding point pairs.

**Figure 29:**
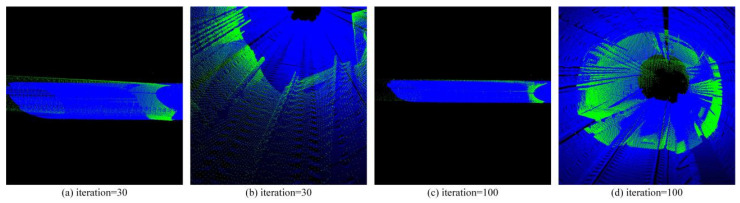
Shows the ICP registration results with different iterations.

**Figure 30:**
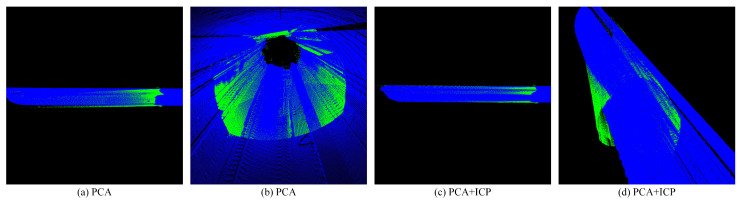
Shows the PCA + ICP registration results.

Then we respectively use the ICP algorithm (Figure 29) and PCA + ICP (Figure 30) algorithm commonly used in registration to conduct experiments on a tunnel point cloud with a length of 50 m.

It can be seen from the final registration results that whether it is the ICP algorithm or the PCA + ICP registration, there will be mismatches, and the result falls into a local optimum, and the matching accuracy rate is low. After the ICP algorithm reaches a certain number of iterations, the accuracy improvement is very limited. Using PCA to perform rough registration in advance will cause the frame point cloud to be reversely registered to the map point cloud, resulting in registration failure.

The algorithm in this paper can not only successfully complete the registration, but also greatly improves the registration accuracy of the more common registration algorithms. It focuses on solving the rapid coarse registration in the subway tunnels, and the correct rate of matching point pairs can reach more than 85%; the root mean square error of registration can reach high-level accuracy. In terms of operation time, since the coarse registration has provided a sufficiently approximate initial value of the pose, the time cost of iteratively solving the optimal transformation in the fine registration is greatly reduced, and finally the registration and positioning with both accuracy and speed are realized.

## 4. Discussion and Conclusions

**Figure 31:**
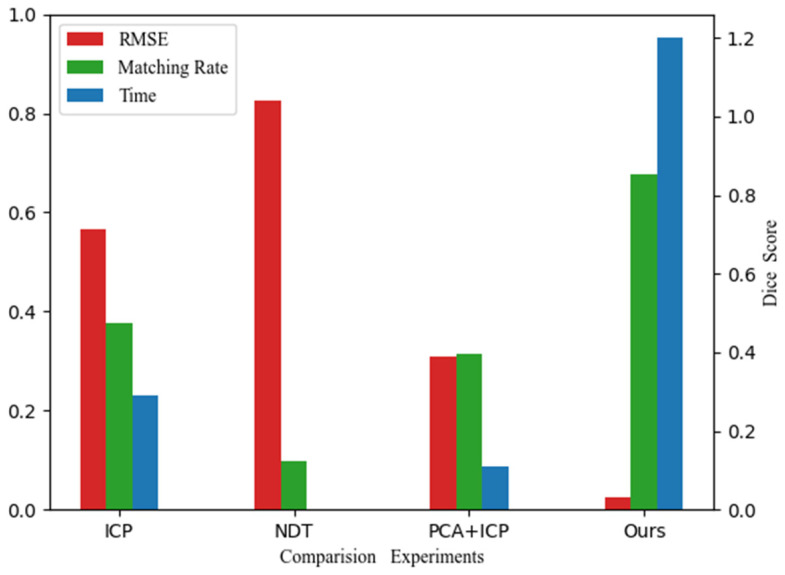
Comparison of registration effects of multiple algorithms by the number of matching point pairs, matching accuracy, RMSE, and time.

In this paper, we study the laser SLAM matching and positioning method for the subway tunnel point cloud. Compared with LOAM and LeGo-LOAM, our coarse-to-fine strategy reduces reliance on high-frequency feature matching, achieving an 85% matching rate (Figure 31). In tunnel environments, Li et al. reported 5 cm accuracy using intensity features, whereas our geometric-convex hull integration achieves 3 cm accuracy, as validated in Section 3.2. In theoretical research, a matching and positioning algorithm is proposed from three aspects: feature extraction, registration strategy, and pose optimization. In practical application, a multi-line lidar data acquisition platform is built, and experimental tests are carried out in the actual tunnel environment. A comprehensive comparison with the common registration algorithm is also carried out. According to the experimental results, we can see that the algorithm has the following characteristics:

- Matching and positioning accuracy is high. Although SLAM can stably perform localization and mapping in most environments, it is prone to tracking loss and drift caused by mismatching in environments with similar spatial structures such as tunnels. The registration algorithm in this paper can directly roughly locate near the correct position, avoiding mismatching, and the subsequent pose optimization algorithm makes a global correction for the still existing errors to ensure the accuracy of positioning in an environment with similar structures.
- The registration efficiency is high. The step-by-step registration strategy adopted by the algorithm in this paper in the coarse registration stage can transform the source point cloud to the approximate position of the target point cloud, which provides a good initial value for fine registration and greatly reduces the amount of calculation in the iterative process. Thus, the registration efficiency is improved.

Overall, the algorithm proposed in this paper can not only register the secondary scanning point cloud of the lidar to the known high-precision map point cloud, and quickly achieve matching and positioning, but also in the case of continuous operation, the pose optimization can be carried out. The algorithm achieves sub-3 cm registration accuracy and 0.7 s processing speed, an 80% improvement in precision and 60% reduction in time compared to ICP and PCA + ICP. Future work will focus on real-time adaptation to dynamic tunnel environments. The coarse-to-fine strategy and keyframe optimization effectively address geometric similarity challenges, making it suitable for autonomous navigation in subway tunnels. It has certain engineering application significance.

## Data Availability

The data that support the findings of this study are available on request from the corresponding author.
